# Combined use of ELISA and Western blot with recombinant N protein is a powerful tool for the immunodiagnosis of avian infectious bronchitis

**DOI:** 10.1186/s12985-018-1096-2

**Published:** 2018-12-12

**Authors:** Paula Fonseca Finger, Michele Soares Pepe, Luana Alves Dummer, Carolina Georg Magalhães, Clarissa Caetano de Castro, Silvia de Oliveira Hübner, Fábio Pereira Leivas Leite, Giseli Aparecida Ritterbusch, Paulo Augusto Esteves, Fabricio Rochedo Conceição

**Affiliations:** 1 Laboratório de Imunologia Aplicada, Centro de Desenvolvimento Tecnológico - Núcleo de Biotecnologia, Pelotas, RS Brazil; 2 Laboratório de Bacteriologia, Centro de Desenvolvimento Tecnológico - Núcleo de Biotecnologia, Pelotas, RS Brazil; 3 Laboratório de Virologia e Imunologia Animal, Faculdade de Veterinária, Pelotas, RS Brazil; 4 Laboratório de Virologia de Aves - Embrapa Suínos e Aves, Concórdia, SC Brazil

**Keywords:** IBV, Nucleoprotein, Recombinant antigen, Diagnosis, Indirect ELISA

## Abstract

**Background:**

The avian infectious bronchitis virus (IBV) remains a significant source of loss in the poultry industry and early diagnosis is required to prevent the disease from spreading. This study examined the combined use of an ELISA and Western blot (WB) to detect antibodies against the nucleocapsid protein (N) of IBV. The coding sequence for N was amplified by RT-PCR and expressed in *Escherichia coli*. A soluble recombinant N protein (rN) of approximately 50 kDa was obtained. A total of 389 sera were tested against the rN in ELISA and the results were compared with those of the commercial IDEXX IBV Ab test. ELISA-rN achieved a 90.34% sensitivity and 90.16% specificity. WB confirmed all false negative sera in ELISA-rN or IDEXX test as truly positive. The current study indicate that the combined use of rN in ELISA and WB is a powerful tool for the immunodiagnosis of avian infectious bronchitis.

**Methods:**

Constructed recombinant pAE/*n* expression vectors were used to transform *E. coli* BL21(DE3) Star competent cells (Invitrogen). The rN of infectious bronchitis virus was purified by affinity chromatography using HisTrap HP 1 mL columns pre-packed with pre-charged Ni Sepharose in the ÄKTAprime Automated Liquid Chromatography system (GE Healthcare). A total of 389 serum samples from chickens were used to develop and evaluate the ELISA-rN test. To standardize the indirect ELISA development, serum dilutions (1:100, 1:200 and 1:400) and different concentrations of purified rN antigen (50, 100 and 200 ng/well) were tested. Positive and negative sera for IBV were used as controls. The results were compared with those obtained from a commercial kit. Serum samples scored as negative with the commercial kit but as positive with the ELISA-rN were further analysed by Western blot analyses using the rN protein as an antigen. The results of the ELISA-rN were compared to the commercial kit results using receiver-operating characteristics curves, area under the curve, and confidence intervals with the software GraphPad Prism version 6.0 for Windows (GraphPad Software, USA).

**Results:**

The expected cDNA fragment of approximately 1240 bp was successfully amplified by PCR using primers designed to select for the coding region of the N protein. The rN was expressed as a soluble protein to avoid the refolding steps and, after purification a yield of 10 mg/L of rN was obtained. The SDS-PAGE results demonstrated the presence of two distinct bands that had a molecular mass of approximately 45 and 50 KDa. Out of 244 sera that scored positive in the commercial ELISA IDEXX IBV Ab Test, 220 were also positive in the ELISA-rN, yielding an ELISA-rN test sensitivity of 90.16%. Out of 145 sera that scored negative in the IDEXX IBV Ab Test, 131 also scored negative in the ELISA-rN, indicating a specificity of 90.34%. Sera that tested negative in the ELISA-rN and positive in the commercial test also reacted with the rN protein in Western blot.

**Conclusions:**

The association between the ELISA and Western blot techniques developed in this study with a subunit of IBV (rN) were able to detect antibodies that the commercial ELISA did not detect suggesting that the ELISA-rN has greater sensitivity.

## Background

Avian infectious bronchitis (IB) is caused by a virus in the *Coronaviridae* family, genera *Gammacoronavirus.* It is a highly contagious disease with a short incubation period [1]*.* The Avian coronavirus was previously classified, and is most commonly referred to, as avian infectious bronchitis virus (IBV). The IBV is responsible for respiratory disease, which manifests in clinical symptoms such as sneezing and tracheal-bronchial rales that can lead to the development of more severe symptoms [2, 3]. Infected birds exhibit reduced performance, consequently leading to a reduction in weight gain and deterioration in egg quality and quantity. Secondary bacterial infections will also contribute to economic losses. Carcass condemnation due to the development of airsacculitis [4, 5] negatively impacts commercial sales of bird meat and eggs. Brazil was once the world’s largest exporter of poultry and currently the world’s third largest producer of bird meat [6]. The consequences of IBV are a significant threat to Brazil’s poultry industry.

The IBV genome consists of a non-segmented positive-sense single-stranded RNA that is approximately 27.6 kb in length. It encodes non-structural (accessory proteins) and four structural proteins: the nucleocapsid protein (N), the spike protein (S), the envelope protein (E), and the matrix protein (M). The nucleocapsid protein, or N protein, consists of 409 amino acids. It has a molecular mass of approximately 50 kDa and directly binds with the viral genome to form the virion nucleocapsid [6, 7]. Its structure is highly conserved, with different strains of IBV sharing a high degree of identity (94–99%) [8]. The N protein is also known for its immunogenicity, inducing specific antibody and cytotoxic T-cells mediated responses [9, 10]. There is significant interest in the use of the IBV N protein as an important target for diagnosis since it possesses the antigenic characteristics required for the development of serological assays that can be applied to detect or quantify antibodies against the IBV [11].

The laboratory diagnosis of IB is dependent on direct and indirect techniques. The direct techniques are employed for viral isolation and genomic or phenotypic identification of the virus, while the indirect methods are used to detect specific antibodies [12]. In addition to being applied for serodiagnosis, serological techniques can also be employed to evaluate the immune responses stimulated by vaccines. Commercial ELISA kits are typically used to indirectly diagnose IBV. These kits, however, are expensive when large number of samples require screening and they are not acessible for applications with the scale of the Brazilian poultry industry [13–15].

ELISA techniques currently available are designed to detect polyclonal antibodies that target the whole virion. The use of nucleoprotein as the antigen for diagnosis and evaluation of vaccine immune responses is an interesting target to explore since this protein plays a important role in IBV virus replication and the induction of a specific immune response in infected birds [16, 17]. The use of recombinant antigens in the design of a specific diagnostic technique facilitates the development of highly sensitive and specific assays that display a high antigen concentration and, thereby, reduce or eliminate background reactions. The use of recombinant antigens also represents a viable method of reducing immunoassay development costs. Easy production of antigens in expression systems leads to simple and efficient antigen development which can reduce the production costs associated with diagnosis [18]. The aim of the current study was to evaluate the combined use of an ELISA and Western blot (WB) to detect antibodies against the nucleocapsid protein of IBV.

## Methods

### Virus strain and viral RNA extraction

A previously characterized Brazilian viral sample of IBV Strain Massachusetts 41 (M41- CNPSA – EMBRAPA – Concórdia, SC, Brazil) was propagated after 9 days of incubation in the chorioallantoic cavity of specific pathogen free (SPF) embryonated chicken eggs. The allantoic fluid was then collected and stored at − 70 °C. Viral RNA extraction was carried out with TRIzol® LS reagent (Invitrogen™, EUA), according to the manufacturer’s instructions.

### N protein coding sequence amplification and cloning

Extracted RNA from IBV strain M41 was used for cDNA synthesis with random oligonucleotides. Reverse transcription (RT) was carried out using SuperScript® One-Step RT-PCR System (Invitrogen, USA). The resulting cDNA samples were used to for PCR amplification of the whole *orf* of the N protein gene. Primers based on the IBV M41 N protein gene sequence available at GenBank (accession number M28566) were designed to align between 102 and 120 and 1312–1331 bp of the gene and include cleavage sites for restriction enzymes. There was a restriction site for *Xho*I in the forward primer (5′ – CCG**CTCGAG**ATGGCAAGCGGTAAGGCAA – 3′) and a restriction site for *Kpn*I in the reverse primer (5′ – GG**GGTACC**TCAAAGTTCATTCTCTCCTA – 3′). The PCR reaction was performed with approximately 25 ng of the extracted cDNA, 3.5 mM MgCl, 0.2 mM dNTPs, 2 units of Taq DNA polymerase, 1X reaction buffer, 1 pmol of each primer, and 5 M N,N,N-trimethylglycine (betaine) under the following conditions: 1 cycle of 95 °C for 7 min, 1 cycle of 70 °C for 1 min, then 45 cycles of 94 °C for 1 min, 50 °C for 1 min, and 72 °C for 4 min, and a final extension of 72 °C for 10 min.

The PCR amplification product was confirmed on a 1% agarose gel and purified using GFX PCR DNA and Gel Band Purification kit (GE Healthcare, Chicago, USA), according to the manufacturer’s instructions.

### Recombinant N protein (rN) expression

The PCR product was cloned into pAE vectors by a T4 DNA ligase (Invitrogen) binding reaction after cleavage with restriction enzymes *Kpn*I and *Xho*I. The constructed recombinant pAE/*n* expression vector was used to transform *E. coli* BL21(DE3) Star competent cells (Invitrogen). The resulting recombinant clones were cultivated in 10 mL of LB broth medium with 100 μg/mL of ampicillin (37 °C, 16 h, 250 rpm). The whole culture volume was transferred to flasks containing 200 mL of LB and incubated at 37 °C with agitation (200 rpm) until the optical density (O.D.) at 600 nm reached 0.8. The expression of the recombinant N protein (rN) was induced by adding isopropyl-β-*d*-thiogalactopyranoside (IPTG, 0.5 mM final concentration) to the culture and incubating for 3 h at 37 °C with agitation (200 rpm). The cells were harvested at 10,000 *x g* for 10 min at 4 °C and the culture pellet containing the rN protein was subjected to a solubilization procedure in ÄKTA wash buffer (0.234% NaH_2_PO_4_, 2.92% of NaCl, 0.068% Imidazole, pH 8.0, supplemented with 20 μg/mL lysozyme). The resuspended cells were submitted to seven sonication cycles of 20 s at 60 Hz and centrifuged again. The rN was purified by affinity chromatography using HisTrap HP 1 mL columns pre-packed with pre-charged Ni Sepharose in the ÄKTAprime Automated Liquid Chromatography system (GE Healthcare). The protein concentration was determined with Qubit™ Protein Assay Kits (ThermoFisher Scientific, USA) according to the manufacture’s instructions.

### Characterization of rN by SDS-PAGE and Western blot

The SDS-PAGE was carried out on 12% polyacrylamide gel. The gels were either stained with Coomassie Blue R-250 (Bio-Rad, California, USA) or electroblotted onto Hybond-ECL 0.45 μm nitrocellulose membranes (GE Healthcare) using the Bio-Rad Mini Trans-Blot Cell (Bio-Rad) for Western blot. Briefly, the membrane was blocked with 5% non-fat milk in phosphate buffer saline containing 0.05% Tween-20 (PBS-T) (137 mM NaCl, 2.7 mM KCl, 100 mM Na_2_HPO_4_, 2 mM KH_2_PO_4_, pH 7.4), incubated with mouse monoclonal antibody (MAb) anti-6xHis (Sigma, USA) diluted to 1:10000 in PBS-T, and incubated with polyclonal antibody Anti-Mouse IgG conjugated to HRP (Sigma). All incubation steps were performed at 37 °C for 1 h under slight agitation followed by three washes with PBS-T. The immunoblot was developed using 3,3′-diaminobenzidine (Sigma).

### Avian serum sample

A total of 389 chicken serum samples (*n* = 389) were used to develop and evaluate the ELISA-rN. The serum samples were kindly provided by MercoLab Laboratories (Garibaldi, Brazil), a laboratory accredited by the MAPA (Ministry of Agriculture, Livestock and Food Supply) that uses the commercial ELISA kit IBV Ab Test (IDEXX) for characterization. Serum samples from chickens with a positive diagnosis for Newcastle disease were used for the evaluation of the ELISA-rN specificity. The samples used in this study originated from birds with a known history of vaccination, including vaccination against the Newcastle disease virus, and were categorized into three groups: commercial egg-layers, broiler breeders, and meat-producing chickens. This information was further used to improve the evaluation of the ELISA-rN test.

### Development of the indirect ELISA-rN

To standardize the indirect ELISA development, serum dilutions (1:100, 1:200 and 1:400) and different concentrations of purified rN antigen (50, 100 and 200 ng/well) were tested. Positive and negative sera for IBV were used as controls. The 96-well microtiter plates (Nunc MaxiSorp®, Thermo Fisher, USA) were coated with 100 ng/well of rN diluted in 0.05 M carbonate-bicarbonate buffer (pH 9.6) and incubated overnight at 4 °C. The plates were incubated at 37 °C for 1 h with a blocking solution (5% non fat dry milk in PBS-T). Serum samples diluted 1:200 in PBS-T were added in duplicate and the plates were again incubated for 1 h at 37 °C. After this period, the secondary antibody horseradish peroxidase (HRP) - rabbit anti-chicken IgY peroxidase conjugate (Sigma) diluted 1:10,000 in PBS-T was added and plates were once more incubated at 37 °C for 1.5 h. After each incubation, the plates were washed three times with PBS-T. In the final step, plates were washed five times and 100 μl/well of peroxidase substrate o-phenylenediamine dihydrochloride (Sigma Aldrich) was added. The reaction was interrupted with 2 N H_2_SO_4_ and the results were read as O.D. using a spectrophotometer at 492 nm. To determine the *cutoff* value, negative sera were used, totalizing 20 sera, considering the mean of sera added of two standard deviation. The ROC analysis was used to simulate the influence of different *cutoff* values on the sensitivity and specificity of the test. The results were compared with those obtained with the commercial kit. Serum that scored as negative in the commercial kit but as positive in the ELISA-rN were submitted to Western blot analyses using the rN protein as antigen.

### Repeatability and specificity of ELISA-rN

In order to evaluate the repeatability of the ELISA-rN, three separate batches of recombinant N protein were produced and purified following the methodology described above with distinct purification times, to demonstrate the reproducibility of the recombinant protein expression procedure. Serum samples were selected for testing against each batch of antigen and the averages, and standard deviations, of the O.D. at 492 nm were calculated. Of the samples used for this test, one was strongly reactive positive, four were moderately reactive positive, and four were negative serum samples.

To evaluate the specificity of the test, negative serum samples to IBV and positive serum samples to Newcastle disease were tested in the ELISA-rN.

### Development of the Western blot-rN

The western blot was performed almost as described above. The rN was deposited in all wells of the SDS-PAGE gel and, after transfer to the nitrocellulose membrane, it was cut to obtain 14 strips. Each strip was incubated with a 1:200 serum dilution after blocking with 5% non-fat milk. The antibody horseradish peroxidase (HRP) - rabbit anti-chicken IgY peroxidase conjugate (Sigma) (1:10000) was added to all membranes and the reaction was revealed using 3,3′-diaminobenzidine (Sigma). All incubation steps were performed at 37 °C for 1 h under slight agitation and were followed by three washes with PBS-T.

### Statistical analysis

The results of the ELISA-rN compared to the commercial kit results, the Receiver-operating characteristics (ROC) curves, the area under the curve (AUC) and the confidence intervals were obtained through the software GraphPad Prism version 6.0 for Windows (GraphPad Software).

## Results

### Cloning and expression of recombinant N protein

The expected cDNA fragment of approximately 1240 bp was successfully amplified by PCR using primers designed to obtain the coding region of the N protein. Successful recombinant pAE/*n* vector construction was confirmed through cleavage with the same restriction enzymes used for the plasmid construction (date not shown).

**Fig. 1:**
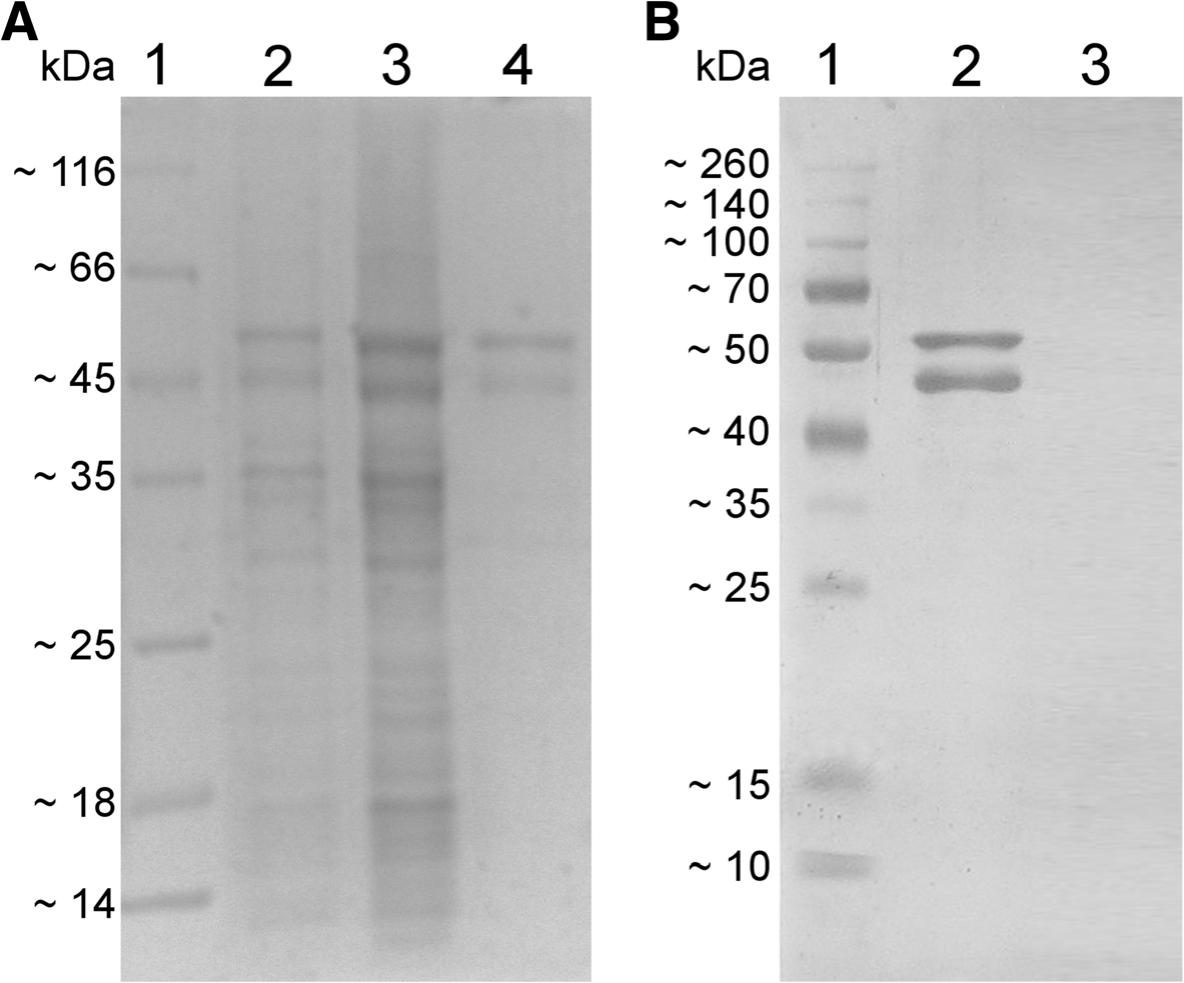
Expression of the recombinant N protein. **a** The rN protein expressed in *E. coli* Unstained MW Marker (ThermoFisher Scientific); Lane 2: Recombinant *E. coli* strain BL21 star (DE3) before protein expression induction; Lane 3: Recombinant *E. coli* strain BL21 star (DE3) after protein expression induction; Lane 4: Purified rN protein. **b** Western blot characterization of the rN protein performed with MAb Anti6xHis. Lane 1: Spectra Multicolor Broad Range Protein Ladder (ThermoFisher Scientific); Lane 2: Recombinant N Protein; Lane 3: Negative control *E. coli* strain BL21 star (DE3)

The recombinant N protein (rN) was expressed as a soluble protein, dispensing refolding steps, and, after purification, a yield of 10 mg/L of rN was obtained. The SDS-PAGE results demonstrated the presence of two distinct bands that had a molecular mass of approximately 45 kDa and 50 KDa (Fig. 1a). The same bands were observed in the Western blot and confirmed the presence of bands that corresponded to the protein of interest (Fig. 1b).

Other studies have also described the cloning of the 1200 bp corresponding to the N protein gene from IBV [18, 19]. They reported that the recombinant protein presented as two distinct molecular masses, one 50 kDa and other 45 kDa. The 45 kDa mass is the truncated form of the N protein [18, 19]. This corroborates with the results observed through SDS-PAGE and Western blot in the current study (Fig. 1).

### Indirect ELISA-rN and ROC analysis

**Fig. 2:**
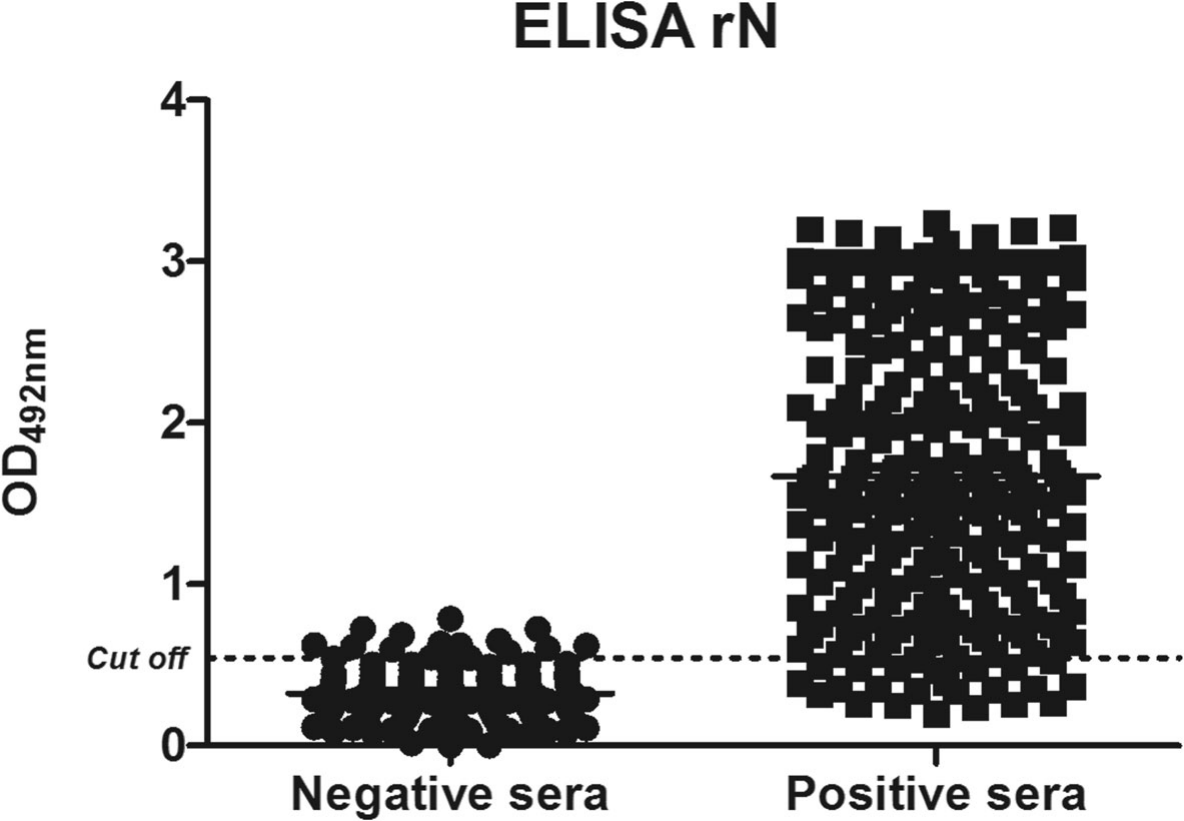
Interactive dot diagram based on ELISA-rN outcomes in relation to ELISA kit IDEXX IBV Ab Test. The results of the commercial test ELISA kit IDEXX IBV Ab Test (IDEXX) were compared with that of the ELISA-rN, confirming 90.16% sensitivity and 90.34% specificity

The results of the ELISA-rN were compared with the results obtained with the IBV Ab Test (IDEXX). Receiver-operating characteristics (ROC) with 95% confidence intervals were used to analyse these results (Fig. 2). Out of 244 sera that scored positive in the IBV Ab Test, 220 also scored positive in the ELISA-rN test. The sensitivity of the ELISA-rN test was 90.16%. Out of the 145 sera that scored negative in the IDEXX Test, 131 also scored negative in the ELISA-rN, indicating a specificity of 90.34%. The area under the curve (AUC) was 0.9588 (*P* < 0.001 and *cutoff* 0.5415).

### Test repeatability and specificity

**Fig. 3:**
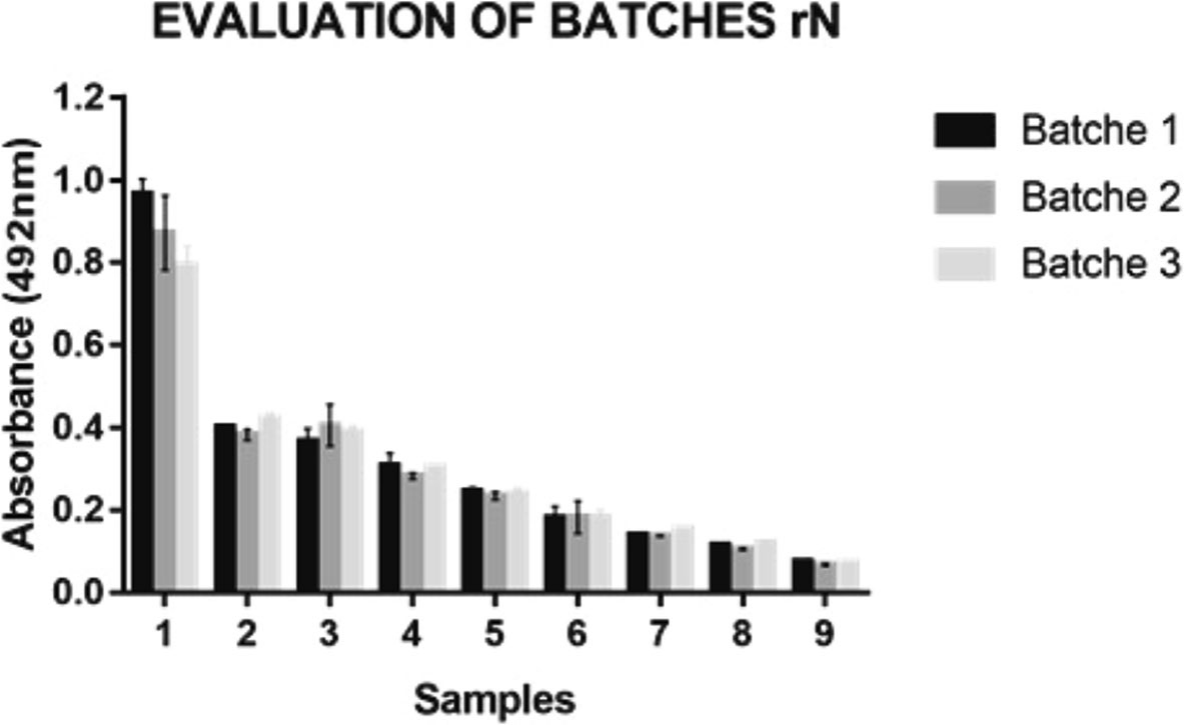
Evaluation of different batches of rN protein production used as antigen in the indirect ELISA development and tested with positive and negative sera for IBV. Sample 1: strongly reactive positive, samples 2–5: moderately reactive positive and samples 6–9: negative serum samples

Three different protein batches were used as coating antigens for testing the ELISA-rN and the results from all three were similar (*P* > 0.05) and, thus, indicate antigen stability (Fig. 3). All Newcastle disease positive sera tested negative in the developed ELISA-rN, providing evidence of test specificity (data not shown).

### Western blot for analysis of discrepant results

**Fig. 4:**
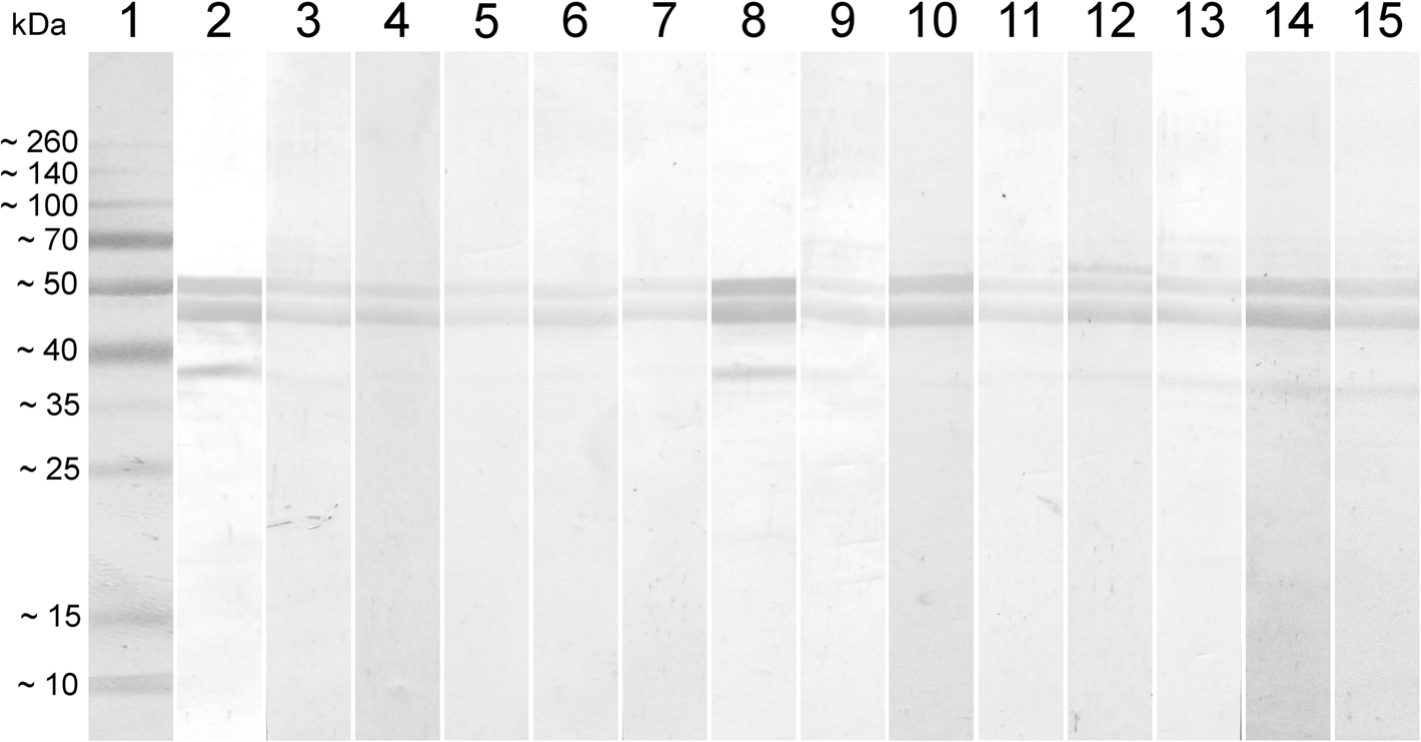
Western blot analyses of the avian serum samples that scored negative for the IDEXX IBV Ab Test and positive in the ELISA-rN. Lane 1: Spectra Multicolor Broad Range Protein Ladder (ThermoFisher Scientific); Lanes 2–15: Serum samples with discrepant ELISA results

All sera that tested negative in the commercial test and positive in the ELISA-rN were submitted for Western blot analyses using the rN protein as an antigen. All serum samples reacted with the two bands of rN, confirming the results obtained in the developed ELISA (Fig. 4). Sera that tested negative in the ELISA-rN and positive in the commercial test also reacted with the rN in WB (data not shown).

## Discussion

Previous studies that focused on the development of more efficient diagnostic techniques were not limited to avian infectious bronchitis detection, but also aimed to control and monitor chickens vaccination [12, 20]. The nucleoprotein from IBV is widely considered the choice protein for the development of immunoassays for antibody detection since this protein plays an important role in inducing an antibody response in IBV infected or vaccinated animals [10, 13]. Other studies have demonstrated that the nucleocapsid protein is highly conserved among IBV isolates (91–96.5% similarity), immunogenic, and abundantly expressed during infection [8, 10, 21]. These features make this protein an interesting candidate for use in diagnostic techniques, such as ELISA and WB [4]. The ELISA is a powerful tool because it provides a safe and an easy way to evaluate the IBV vaccine efficiency, and perform serological diagnosis as well as epidemiological surveillance [18].

In contrast to the ELISA presented in this study, the ELISA developed by Lugovskaya et al. [15] used two fragments of the recombinant N protein expressed in *E. coli* as an antigen, achieved a specificity of 87.36%, and a sensitivity of 93.81%. These results are comparable with the commercial test that is currently used for routine IBV diagnosis that has a specificity of 88.97% and a sensitivity of 92.86% [15]. The ELISA-rN developed in this study presented similar specificity and sensitivity results (90.16 and 90.34%, respectively), and thus highlights that a unique IBV nucleoprotein fragment can be employed to efficiently detect specific antibodies in poultry serum.

Our study uses a Western blot technique to complement the results obtained through ELISA and further support the IBV diagnosis. The Western blot technique may prove useful for epidemiological studies, for monitoring specific pathogen free farms, and in vaccine potency tests. The association of ELISA and Western blot can be seen as a tool to be considered to increase sensitivity for serodiagnosis purposes [22]. Since positive sera react with two bands in the Western blot, the technique is even more specific.

The recombinant N protein produced in this study was expressed in the soluble fraction. Our rN was easily recoverable from the culture without using denaturing agents. In contrast to previous studies where the N protein was expressed in its insoluble form [10, 14], our procedure avoids the necessity to refold the recombinant protein. The production of rN was repeatable since the same ELISA-rN test results, and about the same yield, were obtained when different production and purification times were used.

Besides the use of the N protein for the diagnosis of IBV, an ELISA that was developed using the S protein was also described and compared to a commercial test [23], and achieved a specificity and sensitivity of 89.83 and 92.38%, respectively. However, the N protein offers specific advantages when used for the purpose of diagnosis during viral infection since it is produced in larger quantities than the S protein (the N proteins is produced at a ratio of 6:1 relative to the S protein [24] and plays an important role in the virus’s replications and assembly process [25]. Additionally, the S protein has hypervariable regions that cause mutations in its sequence and therefore offers low efficiency as a protein for diagnosis [1].

The results from the ELISA test developed using the rN protein indicate that the test could be effectively applied for IBV diagnosis [17]. The yield of rN per litre of LB broth culture would coat approximately 1000 96-well microtiter plates allowing for the diagnostic analysis of approximately 45,000 serum samples in duplicate. Also noteworthy is the production cost reduction from using rN in a soluble form, since this eliminates costs associated with the refolding step while it also preserves important conformational epitopes. The currently available commercial test on the other hand, employs the whole IBV as an antigen which requires viral propagation and the implementation of robust laboratory biosafety standards [23].

It is worth mentioning that, by comparing the developed ELISA-rN and WB with the IDEXX IBV Ab Test, the analysis conducted in the current study indicated that it was possible that some serum identified as negative for anti-IBV antibodies on the commercial test be positive when tested using the ELISA-rN and by WB. This could be of concern as false negative results are undesirable, especially if a lot of birds are misdiagnosed. Thus, the ELISA-rN could be applied together with the WB developed in this study for the routine detection of IBV in diagnostic laboratories. False negative results that contribute to the spread of the disease can be avoided, or at least decreased, when the two tests are applied together.

## Conclusion

The indirect ELISA developed here with rN as an antigen allowed for the detection of anti-IBV antibodies in chicken serum at high specificity and sensitivity. The association between ELISA and Western blot techniques developed with a subunit of IBV (rN) were able to detect antibodies that were not detected with the commercial ELISA test suggesting greater sensitivity in the developed ELISA-rN. In addition, the ELISA-rN with the advantages of easy preparation and improved safety could be a promising alternative to the whole live virus ELISA.
